# Computational Assessment of *I*–*V* Curves and Tunability of 2D Semiconductor van der Waals Heterostructures

**DOI:** 10.1021/acs.nanolett.4c06076

**Published:** 2025-01-22

**Authors:** Qiuhua Liang, Samuel Lara-Avila, Sergey Kubatkin, Md. Anamul Hoque, Saroj Prasad Dash, Julia Wiktor

**Affiliations:** † Department of Physics, Chalmers University of Technology, SE-412 96 Gothenburg, Sweden; ‡ Department of Microtechnology and Nanoscience, Chalmers University of Technology, SE-412 96 Gothenburg, Sweden

**Keywords:** Density Functional Theory (DFT) Calculations, Non-Equilibrium Green Function (NEGF), Transition Metal Dichalcogenide (TMD) Heterostructures, Electronic Transport Property, Tunnel Field-Effect Transistors (TFETs)

## Abstract

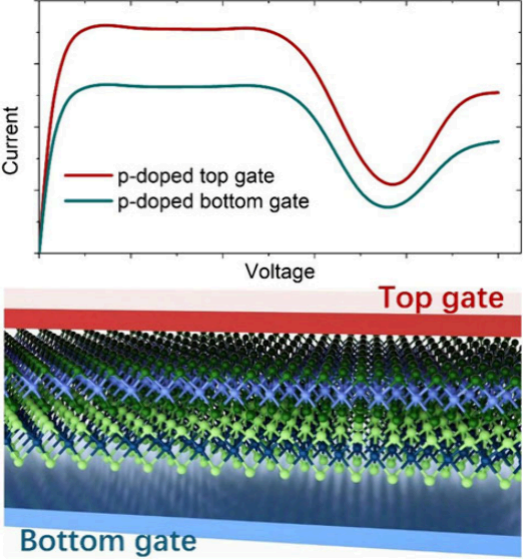

Two-dimensional (2D) transition metal dichalcogenides (TMDs) have received significant interest for use in tunnel field-effect transistors (TFETs) due to their ultrathin layers and tunable band gap features. In this study, we used density functional theory (DFT) to investigate the electronic properties of six TMD heterostructures, namely, MoSe_2_/HfS_2_, MoTe_2_/ZrS_2_, MoTe_2_/HfS_2_, WSe_2_/HfS_2_, WTe_2_/ZrS_2_, and WTe_2_/HfS_2_, focusing on variations in band alignments. We demonstrate that WTe_2_/ZrS_2_ and WTe_2_/HfS_2_ have the smallest band gaps (close to 0 or broken) from the considered set. Furthermore, combining DFT with the nonequilibrium Green’s function method (DFT-NEGF), we analyzed the output *I*–*V* characteristics, revealing increased current as band gap closes across all studied heterostructures. Notably, WTe_2_/ZrS_2_ and WTe_2_/HfS_2_ show a potential negative differential resistance (NDR) even without a broken gap. Importantly, the inclusion of a p-doped gate effect in WTe_2_/ZrS_2_ enhances the current flow and band-to-band tunneling. The rapidly increasing tunneling current under low applied voltage indicates that the WTe_2_/ZrS_2_ and WTe_2_/HfS_2_ heterostructures are promising for applications in TFETs.

Metal-oxide semiconductor field-effect transistors (MOSFETs) have been the cornerstones of modern electronics for decades. However, MOSFETs face significant disadvantages as device dimensions shrink, particularly in high-density integration. One major issue is the high-power dissipation due to heightened leakage currents and, consequently, power losses as devices are scaled down.^1−3^ Additionally, the subthreshold swing limit, governed by the Boltzmann limit, fundamentally challenges the reduction of power consumption.^4−6^ In this context, band-to-band tunnel field effect transistors (TFETs) with low energy consumption can potentially be a compelling alternative to break through the subthreshold swing limit by exploiting quantum tunneling mechanisms instead of thermionic emission.^7−9^

Two-dimensional (2D) transition metal dichalcogenide (TMD) materials have gained widespread attention in the field of nanoelectronics due to their atomic scale thickness, mechanical flexibility, band gap tunability, and high conductivity as alternatives to traditional semiconductor materials.^10−13^ The 2D van der Waals TMD heterostructures (vdWHs), formed by stacking different layers of 2D TMDs, are highly promising for TFETs due to their fascinating properties beyond traditional bulk material limits. For example, the van der Waals interactions facilitate the assembly of various heterostructures from different materials, circumventing the need for lattice matching that is typical in traditional bulk materials with covalent bonds.^14^ Besides, their interfaces are free of dangling bonds and therefore can mitigate the parasitic trap-assisted tunneling current.^15^ Importantly, band alignments of these vdWHs can be effectively manipulated by applying an external electric field or adjusting the interlayer distance.^16−21^ This flexibility offers the possibility of achieving a broken or near-broken gap alignment, an ideal condition that facilitates band-to-band tunneling with minimal energy loss, which is pivotal for the development of TFETs.

Though some broken or near-broken gap 2D vdWHs formed by combining monolayers of, for instance, MoSe_2_, WSe_2_, MoTe_2_, WTe_2_, ZrS_2_, and HfS_2_ have been reported using density functional theory (DFT) simulations,^22−24^ their electronic transport properties remain largely unexplored. Detailed studies on the electronic transport properties of these vdWHs are expected to provide insights into their potential applications in TFETs. In this study, we systematically investigated the electronic and transport properties of vertically stacked MoSe_2_/HfS_2_, MoTe_2_/ZrS_2_, MoTe_2_/HfS_2_, WSe_2_/HfS_2_, WTe_2_/ZrS_2_, and WTe_2_/HfS_2_ vdWHs, because of their similar lattice parameters and reported broken or near-broken band gaps.^22,23,25^ These characteristics facilitate better structural compatibility and potential applicability to TFET applications. Besides, it has been noted that vertical stacking in vdWH TFET results in a smaller gate-drain capacitance compared to lateral vdWH TFET.^26^ We investigated how varying interlayer distances affect the electronic band structures of these vdWHs. Additionally, we systematically analyzed the current–voltage (*I*–*V*) characteristics of these vdWHs at various interlayer distances, utilizing DFT combined with the nonequilibrium Green’s function (DFT-NEGF) calculations. We find that the reduction of the band gap by only 0.03 eV resulted in the increase of the maximum current in the considered voltage range by a factor of 2.62. Interestingly, our findings reveal that the WTe_2_/ZrS_2_ and WTe_2_/HfS_2_ heterostructures exhibit negative differential resistance (NDR) behavior, regardless of variations in the band gap, interesting for multivalued logic (MVL) operation. Additionally, we investigate the effects of the gate voltage on WTe_2_/ZrS_2_. The results indicated that p-type doping can further increase the current and enhance the NDR behavior. This comprehensive study provides a detailed analysis of 2D vdWHs, highlighting their potential for application in TFETs.

Geometry optimizations and electronic structure calculations were performed using the Vienna Ab Initio Simulation Package (VASP) with the projector augmented plane wave (PAW) technique and a plane wave basis set.^27−29^ The generalized gradient approximation (GGA) of Perdew, Burke, and Ernzerhof (PBE) was used for the exchange-correlation effects,^30^ supplemented by Grimme’s DFT-D2 method for van der Waals interactions.^23^ A cutoff energy of 500 eV and a Monkhorst–Pack k-point of 9 × 9 × 1 mesh were employed for high accuracy.

SIESTA was utilized to calculate the electronic properties using norm-conserving Troullier–Martin pseudopotentials,^31,32^ a double-ζ plus polarization (DZP) basis set within the PBE-GGA functional.^30^ This approach was chosen for its ability to provide detailed insights into electronic properties and electron transport calculations, critical to our study.

The electron transport calculations were performed using the DFT combined with the nonequilibrium Green’s function (NEGF) method implemented in TranSIESTA.^33^ The calculations were aimed at understanding the device physics under operational conditions, using the Landauer–Büttiker formula to compute source-drain current.^34,35^ In all of the calculations, spin orbital coupling (SOC) was not included. More details about computation methods can be found in the Supporting Information.

We first relax the monolayers and calculate their electronic band structures. The band structures obtained by using both VASP and SIESTA are given in Figure S1. All the results are in excellent agreement with previously reported theoretical calculations.^22,23,25,36,37^ To explore potential interactions that could lead to new or enhanced electronic properties, we construct six different vdWHs from monolayer TMDs, namely, MoSe_2_/HfS_2_, MoTe_2_/ZrS_2_, MoTe_2_/HfS_2_, WSe_2_/HfS_2_, WTe_2_/ZrS_2_, and WTe_2_/HfS_2_. These vdWHs are developed based on the most stable configurations identified in previous studies,^23−25^ and the strain is uniformly distributed within the layers when stacked. Additionally, all the lattice mismatches in our studied vdWHs are within 5%.^38,39^ The effect of lattice mismatch on the electronic structures of the vdWHs is examined, with band structures calculated using SIESTA and displayed in Figure S3. These results demonstrate that, while lattice mismatch can affect the band structures and band edge positions of the vdWHs, it generally does not alter the overall band alignment. Additionally, the effect of lattice mismatch on the band structure is further analyzed using VASP for the WTe_2_/ZrS_2_ system, as shown in Figure S4. The band structure obtained from VASP closely matches those calculated from SIESTA, further supporting the conclusion that it is reasonable to disregard the impact of lattice mismatch in these calculations. More details about the effect of lattice mismatch on the *I*–*V*_b_ curve will be discussed below. After construction, each vdWH is optimized to determine the most favorable interlayer distances. The optimized interlayer distances, denoted as “*d*”, were 2.96 Å for MoSe_2_/HfS_2_, 3.10 Å for MoTe_2_/ZrS_2_, 3.09 Å for MoTe_2_/HfS_2_, 2.90 Å for WSe_2_/HfS_2_, 3.04 Å for WTe_2_/ZrS_2_, and 3.05 Å for WTe_2_/HfS_2_, as depicted in Figure 1. The *d* of all vdWHs is summarized in Table 1. These values are in good agreement with values reported in the literature, further confirming the reliability of our computational setup.^22−25,40,41^

**Figure 1:**
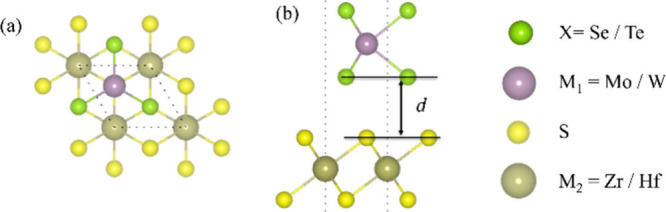
(a) Top and (b) side views of the M_1_X_2_–M_2_S_2_ vdWHs. *d* indicates the interlayer distance.

**Table 1:** Interlayer Distances, *d*, of All vdWHs^a^

vdWHs	MoSe_2_/HfS_2_	MoTe_2_/ZrS_2_	MoTe_2_/HfS_2_	WSe_2_/HfS_2_	WTe_2_/ZrS_2_	WTe_2_/HfS_2_
*d* (Å)	2.96	3.10	3.09	2.90	3.04	3.05
ref.	3.05^40^	3.10^22^	3.14^41^	/	3.10^25^	3.02^23^

a The comparison data from other published works are also listed. “/” indicates that no reference was found.

The band structures of the different vdWHs are plotted in Figure 2 with blue lines. All constructed vdWHs from the monolayer TMDs exhibit indirect band gaps. Specifically, the vdWHs MoSe_2_/HfS_2_, MoTe_2_/ZrS_2_, and MoTe_2_/HfS_2_ have relatively small band gaps, while WSe_2_/HfS_2_, WTe_2_/ZrS_2_, and WTe_2_/HfS_2_ show near-broken band gaps. (In this study, we define a near-broken band gap as a band gap that is smaller than 0.1 eV.) The band gap values are as follows: 0.13 eV for MoSe_2_/HfS_2_, 0.18 eV for MoTe_2_/ZrS_2_, 0.19 eV for MoTe_2_/HfS_2_, 0.04 eV for WSe_2_/HfS_2_, 0.01 eV for WTe_2_/ZrS_2_, and 0.03 eV for WTe_2_/HfS_2_. These values are at the level of accuracy that can be expected when comparing DFT calculations with experiment. Therefore, it is important to assess how variations in the band gap, as could be observed experimentally, would impact the transport properties of the vdWHs. The interlayer coupling in vdWHs, strongly influenced by the interlayer distance, significantly affects the electronic structure and physical properties of these materials. Previous studies have demonstrated that reducing the interlayer distance can effectively modify the values of the valence band maximum (VBM) and conduction band minimum (CBM), as well as change the band gaps.^42^ Additionally, experiments have demonstrated that adjusting the interlayer distance in these vdWHs is possible by applying hydrostatic pressure.^43^ Here we investigated the band structures of these vdWHs at a uniformly reduced *d* value of 2.70 Å. The results of these investigations are depicted in Figure 2, with red lines. Upon reducing *d*, the band gaps of MoSe_2_/HfS_2_, MoTe_2_/ZrS_2_, MoTe_2_/HfS_2_, and WSe_2_/HfS_2_ decreased to 0.06, 0.11, 0.09, and 0.01 eV, respectively, approaching near-broken band gaps. Notably, at a reduced *d* of 2.70 Å, WTe_2_/ZrS_2_ and WTe_2_/HfS_2_ exhibit negative band gaps of −0.03 and −0.004 eV. The negative value indicates an energy overlap of 0.03 and 0.004 eV and is characteristic of a broken gap band alignment. It is particularly noteworthy that, in the cases of MoTe_2_/ZrS_2_, MoTe_2_/HfS_2_, WTe_2_/ZrS_2_, and WTe_2_/HfS_2_, the reduction in interlayer distance not only affects the band gaps but also precipitates a significant shift in the VBM from the K point to the Γ point. These findings reveal that reducing *d* leads to significant changes in the electronic structures of these vdWHs. This alteration profoundly impacts their band alignments and overall electronic properties, demonstrating the sensitivity of these materials to spatial configuration adjustments.

**Figure 2:**
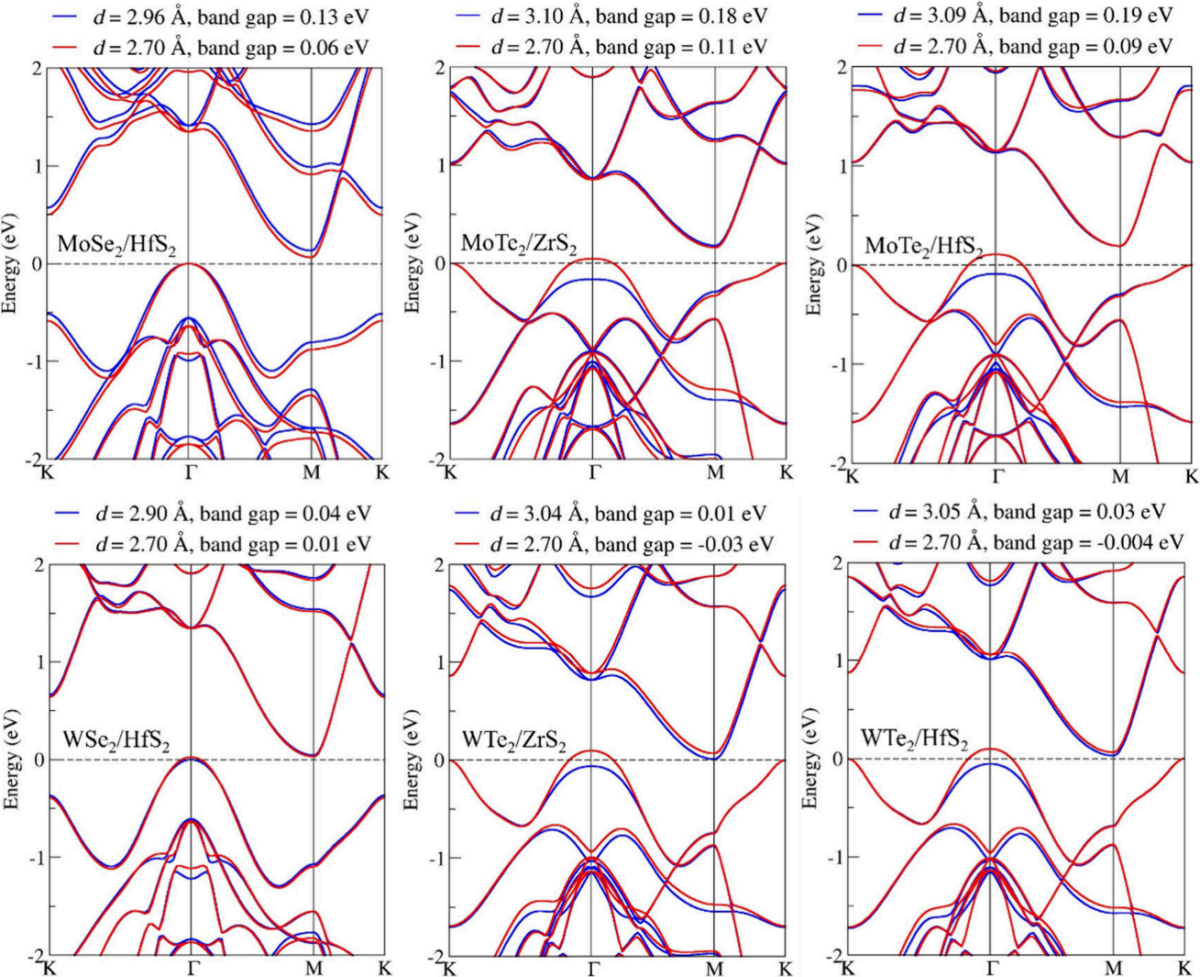
Electronic band structures of vdWHs with different *d* values calculated by SIESTA.

We then qualitatively investigated the quantum transport properties of these vdWHs with both optimized *d* and standardized *d* of 2.70 Å using the DFT-NEGF method. A two-probe model is constructed to mimic the device, which contains a central scattering region and two semi-infinite electrodes (source and drain), as shown in Figure 3. The entire modeled system contains 336 atoms, with the quantum transport direction occurring between the left and right electrodes. Previous theoretical studies have validated the use of simplified device models to investigate transport properties, confirming their reliability.^25,44−46^

**Figure 3:**
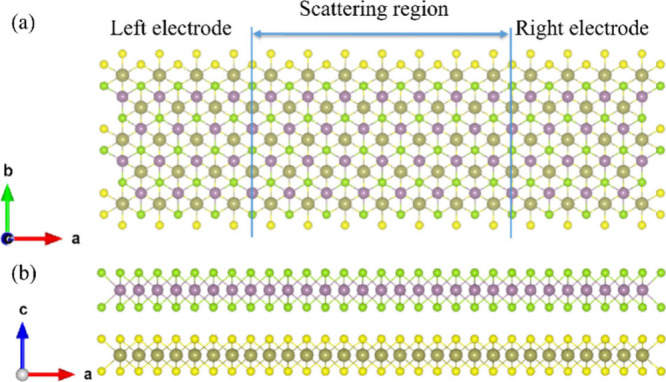
(a) Top and (b) side views of the transport setup with a left electrode, a central scattering region, and a right electrode.

Figure 4 shows the calculated output (*I*–*V*_b_) characteristics for these vdWHs at both an optimized *d* and a uniform *d* of 2.70 Å. Notably, WTe_2_/ZrS_2_ exhibits the highest current across all applied voltages among the vdWHs, as illustrated in Figure 4(a). This is followed by WSe_2_/HfS_2_ and WTe_2_/HfS_2_. Specifically, WTe_2_/ZrS_2_ shows a rapid increase to a high current of 0.54 μA from 0 to 0.1 V. The current then levels off, forming a plateau from approximately 0.1 to 0.5 V, before descending to a valley of 0.34 μA at 0.8 V. The peak-to-valley ratio for this structure is calculated to be 1.59, which is larger than the reported value of 1.00 (1.08) in phosphorene/SnS_2_ (SnSe_2_) vdWHs.^46^ Beyond 0.8 V, the current began to rise again. A similar trend is observed in the WTe_2_/HfS_2_ vdWH, but with a lower peak value of about 0.37 μA at 0.1 V and valley value of 0.26 μA at 0.7 V, resulting in a peak-to-valley ratio of 1.42. Both WTe_2_/ZrS_2_ and WTe_2_/HfS_2_ vdWHs display NDR behavior, and the appearance of NDR confirms the presence of band-to-band tunneling (BTBT) transport mechanisms within these structures. In contrast, WSe_2_/HfS_2_ maintains a steady current of about 0.37 μA across most of the voltage range, demonstrating a stable electronic response. MoTe_2_/ZrS_2_ remains relatively flat with small current values from 0 to 0.5 V but exhibits a rapid increase after 0.5 V, reaching approximately 0.51 μA at 1.0 V. MoSe_2_/HfS_2_ and MoTe_2_/HfS_2_ consistently exhibit very low currents compared to the others. For MoSe_2_/HfS_2_, MoTe_2_/ZrS_2_, MoTe_2_/HfS_2_, and WSe_2_/HfS_2_, the NDR behaviors are not observed.

**Figure 4:**
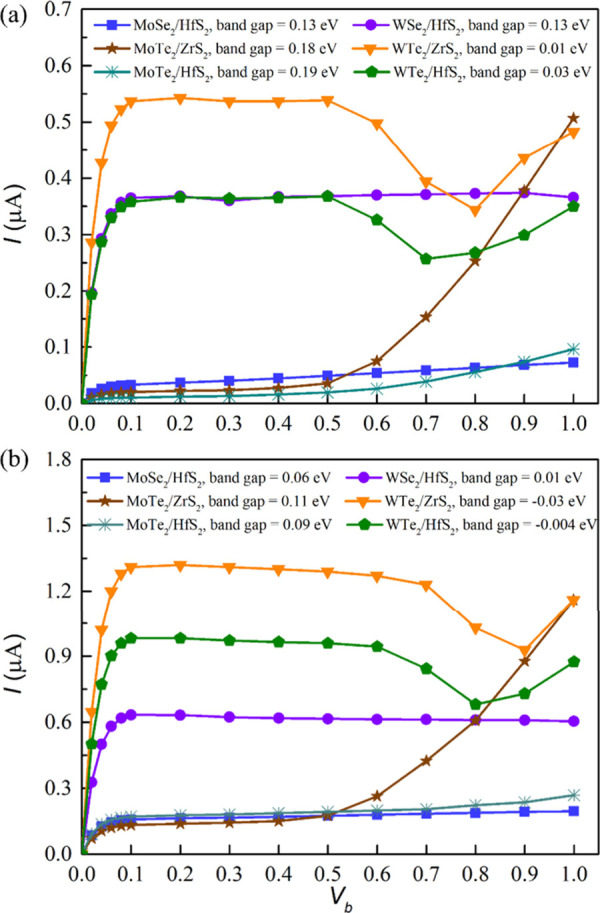
*I*–*V*_b_ curves of the heterostructures with (a) optimized *d* and (b) *d* = 2.70 Å.

To understand why tungsten-based vdWHs show higher currents than molybdenum-based vdWHs, we perform an analysis of the Bader charge transfer. As shown in Table S1, the overall charge transfer is small due to the weak van der Waals interaction. Nevertheless, tungsten-based vdWHs consistently display charge transfers larger than those of the corresponding molybdenum-based vdWHs. In other words, substituting Mo with W increases the net charge transferred between layers, which is generally associated with stronger electrostatic interactions. Consequently, this enhanced charge transfer boosts the carrier concentration at the interface and thus tailoring of the electronic transport along the interface,^47^ thereby improving electrical conductivity and leading to higher current.

Besides, a deeper insight into the observed *I*–*V*_b_ characteristics of MoTe_2_/ZrS_2_ and WTe_2_/ZrS_2_ vdWHs at optimized *d* has been analyzed in terms of the behavior of the transmission coefficient. The current in MoTe_2_/ZrS_2_ rises steadily with increasing bias from 0.5 to 0.9 V because more transmission channels enter the bias window, as shown in Figure S5. Conversely, WTe_2_/ZrS_2_ shows a decrease in current at bias from 0.5 to 0.8 V as its active transmission channels move away from the most relevant energy range, followed by a current increases again at 0.9 V when a new transmission peak appears. More details are discussed in the Supporting Information.

To examine the effect of lattice mismatch on the *I*–*V*_b_ characteristics, we take WTe_2_/ZrS_2_ as a case study and calculate its *I*–*V*_b_ with 5% compression in the lattice. The results, displayed in Figure S6, indicate that compressing the lattice constants leads to a larger current than the uncompressed system at the same applied voltage. The maximum current reached approximately 0.98 μA between 0.1 and 0.3 V, and the minimum current was about 0.62 μA at *V*_b_ = 0.7 V. Despite the compression, the system still exhibits a similar trend to the uncompressed setup, displaying NDR behavior with a peak-to-valley ratio of 1.58. Thus, these findings suggest that the lattice mismatch does not alter our initial conclusions.

After reducing *d*, and therefore the band gaps, the vdWHs show similar current trends as observed with optimized *d*, as illustrated in Figure 4b. Notably, Figure 4b generally shows higher current outputs across all vdWHs compared with Figure 4a. Specifically, WTe_2_/ZrS_2_ demonstrates a sharp increase from 0 to 0.1 V with a peak value of about 1.32 μA, maintaining a plateau from approximately 0.1 to 0.7 V, before reaching a valley value of 0.93 μA at 0.9 V. The peak-to-valley ratio is calculated to be 1.42. NDR behavior is observed when *V*_b_ exceeds 0.6 V and dissipates above 0.9 V. For WTe_2_/HfS_2_, the current also increases rapidly from 0 to 0.1 V, achieving a maximum of about 0.97 μA, with a plateau extending from around 0.1 to 0.6 V, and reaching a valley value of 0.68 μA at 0.8 V. NDR behavior is similarly noted for *V*_b_ beyond 0.6 V and is lost after 0.8 V, with a peak-to-valley ratio of 1.43.

The other structures, MoSe_2_/HfS_2_, MoTe_2_/ZrS_2_, MoTe_2_/HfS_2_, and WSe_2_/HfS_2_, still do not exhibit NDR behavior but show higher current outputs compared to those at optimized *d*. Specifically, WSe_2_/HfS_2_ maintains a steady current of about 0.62 μA across most of the voltage range. The *I*–*V*_b_ curve of MoTe_2_/ZrS_2_ remains relatively flat with low current values from 0 to 0.5 V but experiences a notable rise after 0.5 V, reaching about 1.16 μA at *V*_b_ of 1.0 V. Both MoSe_2_/HfS_2_ and MoTe_2_/HfS_2_ continue to exhibit low currents throughout the entire *V*_b_ range compared to the other structures.

The observed increase in current, consistent with the decreases in the band gaps of the vdWHs, reflects the significant influence of interlayer compression on the electronic transport properties. Interlayer compression may enhance interlayer coupling in vdWHs, thus leading to a reduction in the band gaps and an increase in current. The exceptional transport performance of WTe_2_/ZrS_2_ and WTe_2_/HfS_2_ vdWHs, characterized by rapid current increases within 0.1 V and evident NDR behaviors, highlights their potential for application in TFETs.

Finally, we simulated the effect of gate voltage on the transport property of WTe_2_/ZrS_2_ at optimized *d* by introducing a specific fixed charge in the system, ensuring the total charge in the system remained zero.^48−50^ Both positive (p-type) and negative (n-type) doping are considered, with a doping concentration of 1.39 × 10^13^ cm^–2^.^50^ For each type of doping, both the top gate and bottom gate are analyzed, respectively, as depicted in Figure 5a.

**Figure 5:**
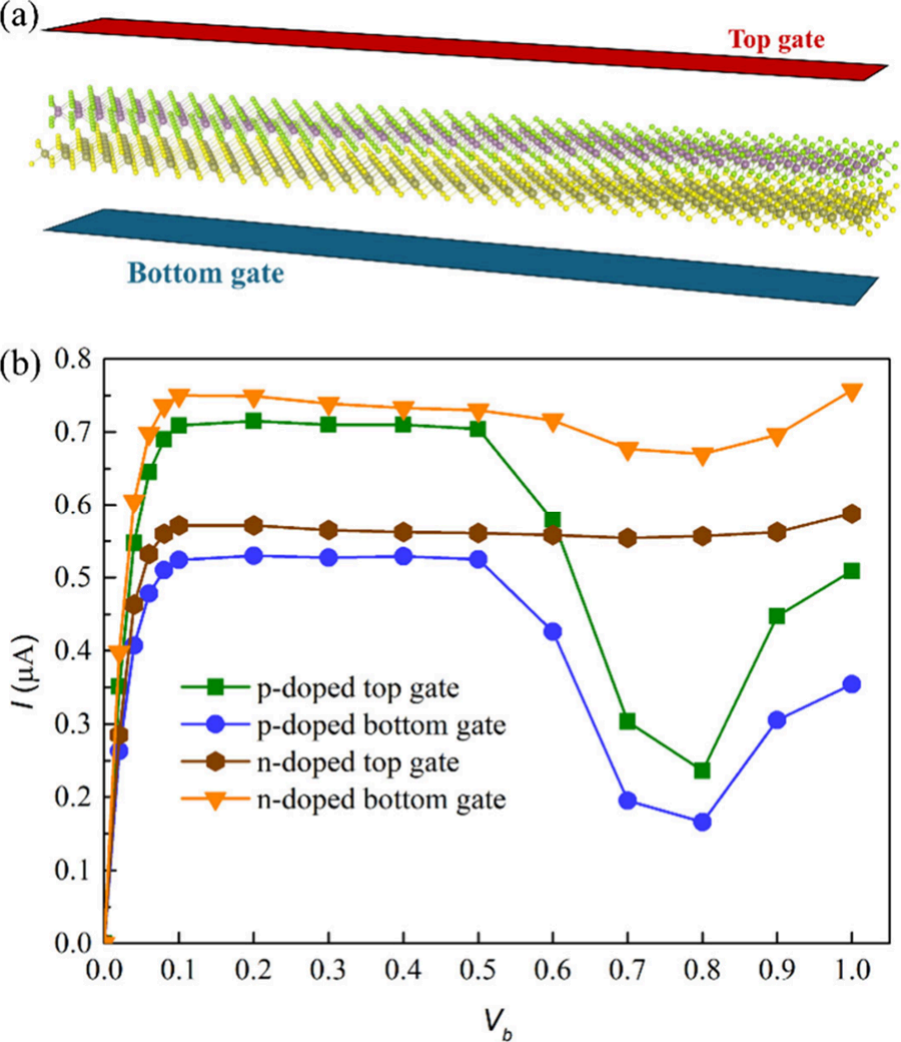
(a) Schematic diagram of applying the bottom gate and top gate on the device and (b) *I*–*V*_b_ curves of p-doped and n-doped WTe_2_/ZrS_2_ at the optimized *d*.

In Figure 5b, we present the calculated *I*–*V*_b_ curves for both p-type and n-type doping at the top gate and bottom gate configurations. After doping, the current can further increase compared to the undoped system. And like the undoped WTe_2_/ZrS_2_ system, the *I*–*V*_b_ curves of the p-doped WTe_2_/ZrS_2_ system show evident NDR behavior. Specifically, in the p-type doped top gate system (green line), there is an enhanced current compared to the undoped system, with the current increasing sharply from 0 to 0.1 V and peaking at 0.71 μA, before reaching a valley of 0.24 μA at 0.8 V. NDR behavior is observed for *V*_b_ > 0.5 V and disappears above 0.8 V. The peak-to-valley ratio increases to 2.96, which is comparable to the recent experimental value of approximately 3 found in MoTe_2_/SnS_2_.^21^ In the p-type doped bottom gate system (blue line), the current also increases sharply from 0 to 0.1 V with a peak of 0.52 μA and reaches a valley of 0.17 μA at 0.8 V. Here too, NDR behavior is evident for *V*_b_ > 0.5 V and vanishes above 0.8 V. The peak-to-valley ratio in this configuration is calculated to be 3.06. The larger peak-to-valley ratio in both p-doped systems compared to the undoped system suggests that more pronounced BTBT behavior may occur in the p-doped system.

Conversely, the n-doped systems do not exhibit significant or any NDR behavior. In the n-type doped top gate system (brown line), the current rises to 0.57 μA at *V*_b_ = 0.1 V and maintains a steady value at *V*_b_ > 0.1 V, with no NDR behavior observed. In the n-type doped bottom gate system (orange line), the current peaks at 0.75 μA at *V*_b_ = 0.1 V and slightly decreases to 0.67 μA at *V*_b_ = 0.8 V, before increasing again after *V*_b_ > 0.8 V, with a peak-to-valley ratio of 1.12; the NDR behavior here is not as evident as in the undoped and p-doped systems. When applying different gate doping, the different interlayer polarizations and the electric field direction across the semiconductor channel may result in the different *I*–*V*_b_ characteristics.^51^ The peak-to-valley ratios of WTe_2_/ZrS_2_ without and with a gate voltage effect are summarized in Table 2.

**Table 2:** Peak-to-Valley Ratio of WTe_2_/ZrS_2_ without and with the Gate Voltage Effect

WTe_2_/ZrS_2_	without gate effect	p-doped top gate	p-doped bottom gate	n-doped top gate	n-doped bottom gate
peak-to-valley ratio	1.59	2.96	3.06	1	1.12

Besides, we investigated the effect of a p-doped bottom gate on WTe_2_/ZrS_2_ as a case study to assess how gate strength influences the peak-to-valley ratio in the NDR behavior. Concentrations of 1.39 × 10^13^ and 0.56 × 10^13^ cm^–2^ are shown in Figure S7. The results demonstrate that the strength of the gate effect can significantly affect the peak-to-valley ratio. Lower doping concentration results in a lower maximum current. Specifically, the system with 0.56 × 10^13^ cm^–2^ exhibits a peak-to-valley ratio of 2.08, which is lower than the 1.39 × 10^13^ cm^–2^ system with a peak-to-valley ratio of 3.06. Thus, a stronger gate effect results in a higher maximum current and a higher peak-to-valley ratio.

We note that our calculations do not include the SOC. This effect will result in the splitting at the VBM of TMDs.^52^ To determine the effect of SOC on the electronic structures of vdWHs, we chose WTe_2_/ZrS_2_ as a case study. The band structures were calculated using PBE and PBE0, with and without SOC, performed in VASP and displayed in Figure S8. Due to error cancellation, we find that PBE and PBE0+SOC exhibit the same extent of the broken gap. Considering the accuracy of the band gap and the significantly increased computational demands of using PBE0+SOC, we opted not to include SOC in our primary calculations and used PBE instead. More details are available in the Supporting Information.

Overall, the p-doped top gate system shows enhanced current and NDR behavior, the p-doped bottom gate system shows enhanced NDR behavior, and the n-type doped systems primarily show enhancements in current but not NDR behavior. These findings highlight the critical influence of the gate voltage on the electronic transport properties of vdWHs, providing valuable insights into the design and optimization of TFETs.

In conclusion, we combined density functional theory (DFT) with nonequilibrium Green’s function (NEGF) methods to investigate the electronic and transport properties of MoSe_2_/HfS_2_, MoTe_2_/ZrS_2_, MoTe_2_/HfS_2_, WSe_2_/HfS_2_, WTe_2_/ZrS_2_, and WTe_2_/HfS_2_ heterostructures. We assessed the effects of reducing interlayer distances on the band structures of these heterostructures, highlighting the reduction of band gaps and the emergence of broken gaps in WTe_2_/ZrS_2_ and WTe_2_/HfS_2_. The *I*–*V* characteristics calculated for these heterostructures show increased current with a decreased band gap. Notably, WTe_2_/ZrS_2_ and WTe_2_/HfS_2_ demonstrate sharp increases in current within low applied voltage and the negative differential resistance (NDR) behavior across various distances. A slight reduction in the band gap by just 0.03 eV results in an increase in the maximum current within the considered voltage range—by factors of 2.44 and 2.62 for WTe_2_/ZrS_2_ and WTe_2_/HfS_2_, respectively. Importantly, we also considered the gate effect and found that the p-doped gate effect can further increase the current and enhance the band-to-band tunneling behavior. Our findings suggest that achieving a broken band gap in heterostructures is challenging; however, minimizing the band gap, even if it is not fully broken, remains a crucial objective due to its potential to enhance electronic properties.

## Supporting Information Available

Computational details, band structures calculated by VASP and SIESTA, k-point test results (WSe_2_/HfS_2_), lattice mismatch effects (vdWHs, SIESTA), band structures (WTe_2_/ZrS_2_, +5% and no compression, VASP), Bader charge analysis (all vdWHs), transmission coefficients (MoTe_2_/ZrS_2_, WTe_2_/ZrS_2_, various voltages), *I*–*V* curves (WTe_2_/ZrS_2_, +5% and no compression, p-doping), and comparison of hybrid functional and SOC effects on band structure (WTe_2_/ZrS_2_) (PDF)
