# Supplementary material for: Computational Assessment of I–V Curves and Tunability of 2D Semiconductor van der Waals Heterostructures
Source: Nano Lett. 2025 Jan 22;25(5):2052–8. doi: 10.1021/acs.nanolett.4c06076 (PMC11803710; doi:10.1021/acs.nanolett.4c06076)
Supplement: Supplementary file 1 — nl4c06076_si_001.pdf [file nl4c06076_si_001.pdf]

Computational assessment of I-V curves and tunability of 2D semiconductor van der Waals heterostructures

Qiuhua Liang[1], Samuel Lara-Avila[2], Sergey Kubatkin[2], Md. Anamul Hoque[2], Saroj Prasad Dash[2], Julia Wiktor[1*]*

1. Department of Physics, Chalmers University of Technology, SE-412 96 Gothenburg, Sweden

2. Department of Microtechnology and Nanoscience, Chalmers University of Technology, SE-412 96 Gothenburg, Sweden

*qiuhua.liang@chalmers.se

*julia.wiktor@chalmers.se

Computational Methods

All the geometry optimizations were performed by Vienna Ab Initio Simulation Package (VASP), which uses the projector augmented plane wave (PAW) technique, and a plane wave basis set.[1-3] The generalized gradient approximation (GGA) of Perdew, Burke, and Ernzerhof (PBE) were utilized for the exchange-correlation effects.[4] The valence electron wavefunctions were expanded in a plane-wave basis with an energy cutoff of 500 eV to ensure convergence of the results. A Monkhorst-Pack k-point mesh of 7 × 7 × 1 and 9 × 9 × 1 were used for geometry optimization and static electronic structure calculations, respectively. The convergence criteria for the forces were set at 0.01 eV/Å and the energy convergence criterion was set at 10^{-5} eV to achieve precise energy minimization. Additionally, to account for the weak van der Waals interactions, we incorporated the DFT-D2 method of Grimme, following the methodology in similar studies.[5-6] To avoid the interactions of adjacent slabs, a vacuum spacing of 30 Å was introduced along the z-direction to eliminate any spurious interactions between periodic images.

SIESTA computations were conducted to calculate the electronic property using

norm-conserving pseudopotentials generated via the Troullier–Martin method.[7-8] The exchange-correlation potential was approximated by the PBE-GGA functional.[4] The k-point sampling for the Brillouin zone integration was denser at 10 × 10 × 1 to enhance the precision of electronic property calculations. The density matrix convergence criterion was set at 10^{-5} and the force tolerance was specified as 0.02 eV/Å. The valence electron wavefunctions were described using a double-ζ plus polarization (DZP) basis set with an energy cutoff of 300 Ry. SIESTA code was based on the linear combination atomic orbital (LCAO) method and uses a basis set consisting of numerical atomic orbitals (NAOs), which were localized functions centered on the atoms. The choice of basis set was crucial as it influences both the accuracy and efficiency of the simulations. To achieve better approximations of the wavefunctions and more accurate results, optimization of the basis set was implemented. The electronic band structures of monolayers $MoSe_2$, $MoTe_2$, WSe_2, WTe_2, ZrS_2, and HfS_2 by utilizing both plane-wave and LCAO within DFT calculations were discussed in **Figure S1**. This was particularly significant for enhancing the accuracy of electron transport calculations. In all the calculations, spin orbital coupling (SOC) was not included, the effect of SOC on the band structure was shown in **Figure S8**, with further details discussed below.

The electron transport calculations were performed in the framework of DFT combined with non-equilibrium Green's function (NEGF) method, which is implemented in the TranSIESTA.[9] The same basis sets and cutoff energy as for the electronic calculations were employed for the transport calculations, and the electronic temperature for the simulations was set at 300 K. A 50 × 10 × 1 k-point mesh was employed for the elongated cell and the 50 k-points were along the transport direction. To ensure the accuracy of our simulations while optimizing computational resources, we tested a denser 100 × 20 × 1 k-point mesh (see **Figure S2**) and compared the results with those from a 50 × 10 × 1 k-point mesh. The comparison revealed that the 50 × 10 × 1 k-point mesh is sufficient to maintain accuracy in the results. In our calculations, the electron-phonon scattering effect was not considered, corresponding to the so-called ballistic approach. At T = 300 K, the thermal energy (k_BT) is about 26 meV, which is

comparable to or lower than the energies of many optical phonons in TMDs. The electron-phonon scattering rate decreases at lower temperatures, and is significantly smaller than typical phonon energies, as fewer phonons are thermally excited. However, we acknowledge that non-thermal mechanisms, such as Fröhlich coupling with optical phonons, could still contribute to scattering. However, our choice to use a ballistic transport model stems from its ability to provide a clear, intrinsic understanding of the transport properties in vdW heterostructures without the additional complexity introduced by dissipative mechanisms. Additionally, we have demonstrated that the I-V curves (specifically the maximum current) are most sensitive to the size of the band gap. A previous study[10] by some of the authors has conducted molecular dynamics (MD) simulations at room temperature to assess the thermal effects on the band alignment of a vdW heterostructure. The results have shown that the band alignment, a critical factor determining the tunneling characteristics, remains stable under thermal effects. This suggests that the ballistic model can provide meaningful insights into the transport behavior of these heterostructures under idealized conditions.

The source-drain current was calculated by the Landauer–Büttiker formula:[11-12]

$$I(V_b) = \frac{2e}{h}\int_{\mu_R}^{\mu_L} T(E,V_b)(f(E-\mu_L) - f(E-\mu_R))dE \qquad (1)$$

where e and h are the electron charge and Planck ’ s constant, respectively. The functions $f(E-\mu_{L(R)})$ is the fermi distribution function of left (right) electrode, $\mu_{L(R)}$ is the chemical potential in the left(right) electrode. The electrochemical potential difference between the left and right electrodes is $eV_b = \mu_L - \mu_R$. The term $T(E,V_b)$ represents the transmission probability of electrons through a device at energy E under potential bias voltage V_b, which is given by the formula:

$$T(E,V_b) = Tr[I(\Sigma_L)G^r I(\Sigma_R)G^a](E,V_b) \qquad (2)$$

where $G^{r(a)}$ is the retarded (advanced) nonequilibrium Green ’ s functions of the scattering region, $\Sigma_{L(R)}$ is the self-energy of the left/right electrode.

Comparison of electronic band structures of monolayer TMDs calculated by VASP and SIESTA

We first relaxed the monolayer TMDs unit cells, the in-plane lattice parameters of H-phase $MoSe_2$, $MoTe_2$, WSe_2, WTe_2, and T-phase ZrS_2 and HfS_2 are 3.32, 3.53, 3.30, 3.55, 3.65 and 3.64 Å, respectively, which are in excellent agreement with previously reported theoretical calculations.[5-6, 13-14] We then calculated the electronic band structures of monolayer $MoSe_2$, $MoTe_2$, WSe_2, WTe_2, ZrS_2, and HfS_2. The band structures obtained using both VASP and SIESTA are displayed in **Figure S1**. This comparison aims to ensure that the LCAO method provides reliable and consistent data, aligning closely with the widely accepted plane-wave calculations, which are known for their precision in electronic structure analysis. The band gaps calculated using SIESTA for $MoSe_2$, $MoTe_2$, WSe_2, WTe_2, ZrS_2, and HfS_2 are 1.44 eV, 1.14 eV, 1.61 eV, 1.12 eV, 1.26 eV, and 1.30 eV, respectively. These values are closely aligned with those obtained by VASP, which yielded band gaps of 1.44 eV, 1.14 eV, 1.62 eV, 1.06 eV, 1.11 eV, and 1.31 eV for the same materials, respectively. These values are consistent with previously published data on the corresponding materials.[5-6, 13, 15] Additionally, the key aspects of the band structures obtained using SIESTA closely resemble those obtained with VASP. This similarity is particularly evident across the high symmetry points within the Brillouin zone. The consistency between the results from both methods validates the accuracy of SIESTA for modeling the electronic structures of these materials. Therefore, subsequent electronic property calculations are performed using SIESTA.

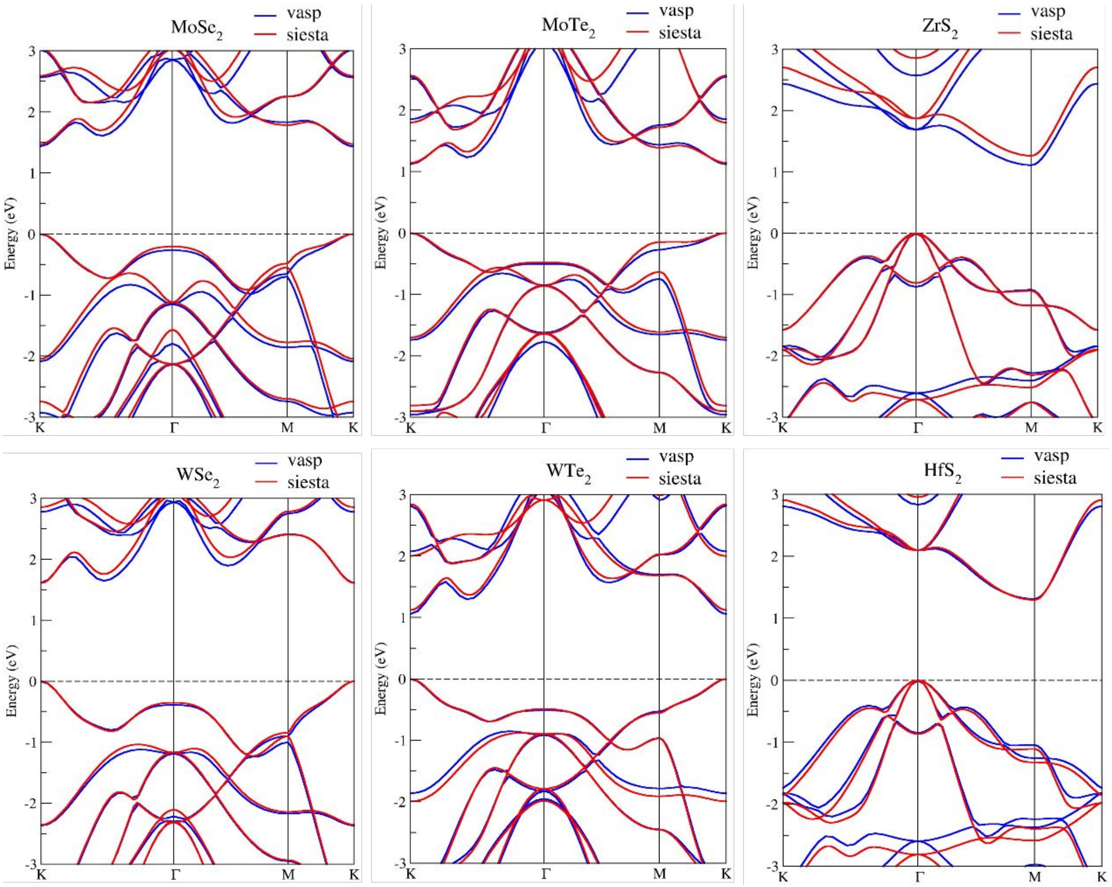

Figure S1. Comparison of the electronic band structures of monolayer $MoSe_2$, $MoTe_2$, WSe_2, WTe_2, ZrS_2, and HfS_2 calculated by VASP and SIESTA.

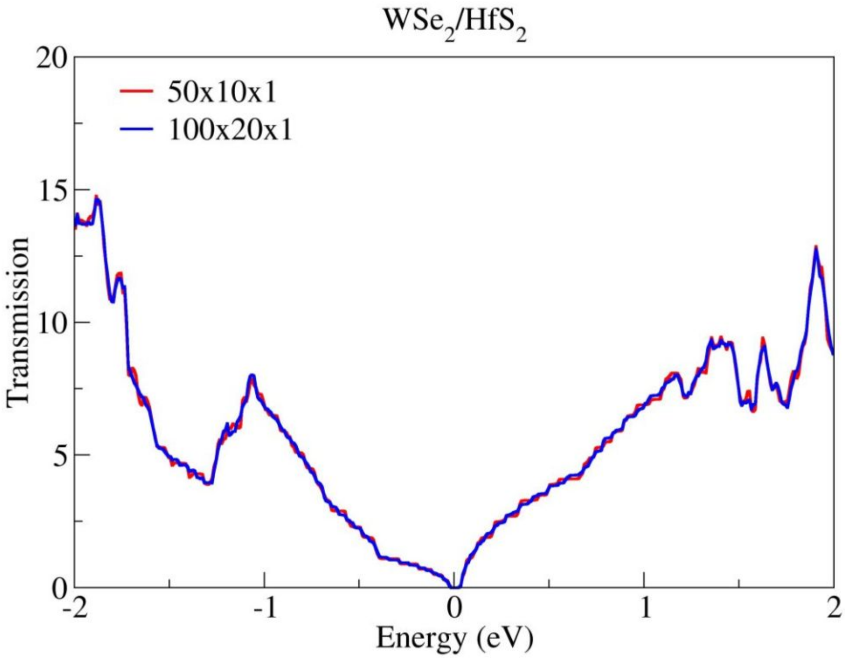

Figure S2. Transmission coefficient versus energy at V_b = 0.02 V of the WSe_2/HfS_2 vdWH for the K-point test.

We also examine the effect of lattice mismatch on the electronic band structures of vdWHs. **Figure S3** shows the band structures calculated by SIESTA without strain, (i.e. the lattice parameters after constructing the vdWHs), and with 5% compression. Compression notably affects the band structures, particularly at high symmetry points. For all vdWHs, the conduction band minimum (CBM) at the M point slightly shifts downward, while the valence band maximum (VBM) at the gamma point shifts upward. However, the overall band alignment remains unchanged. Additionally, we used WTe_2/ZrS_2 as a specific case to observe how compression affects the band structures, with calculations performed using VASP, as shown in **Figure S4**. The band structure results from VASP closely match those from SIESTA, further validating the observed effects.

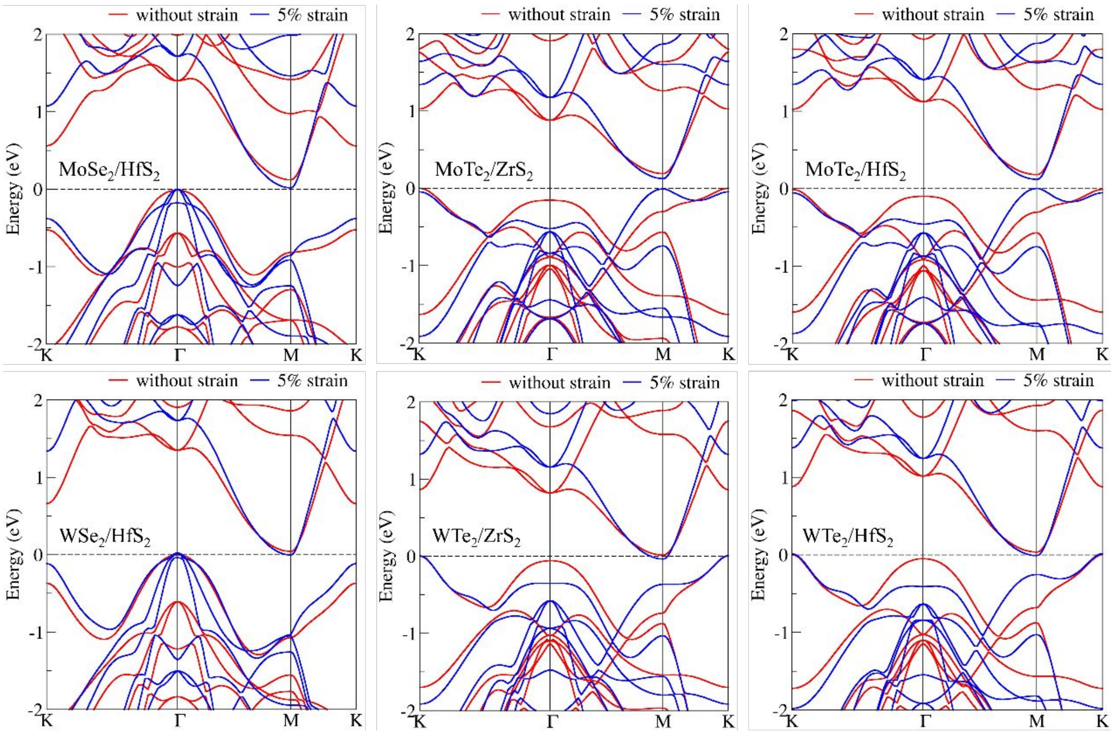

Figure S3. Comparison of the effect of lattice mismatch on the vdWHs with no lattice mismatch and with 5% compression, calculated by SIESTA.

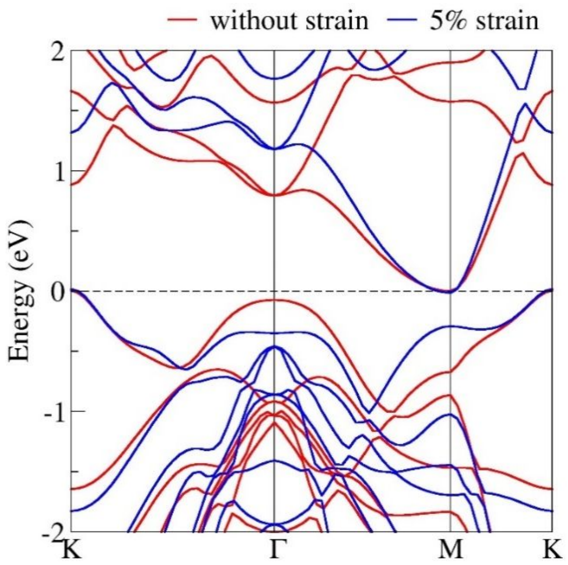

Figure S4. Comparison of the effect of lattice mismatch on WTe_2/ZrS_2 with no lattice mismatch and with 5% compression, calculated by VASP.

To understand why tungsten-based vdWHs show higher currents than molybdenum-based vdWHs, we perform an analysis of the Bader charge transfer within VASP. **Table S1** Shows the amount of transferred charge for each heterostructure.

Table S1. Calculated Bader charges (in *e* per unit cell) of vdWHs.

Systems	Obtained	Lost	Charge transfer
$MoSe_2/HfS_2$	HfS_2	$MoSe_2$	0.015
WSe_2/HfS_2	HfS_2	WSe_2	0.021
$MoTe_2/ZrS_2$	ZrS_2	$MoTe_2$	0.043
WTe_2/ZrS_2	ZrS_2	WTe_2	0.048
$MoTe_2/HfS_2$	HfS_2	$MoTe_2$	0.034
WTe_2/HfS_2	HfS_2	WTe_2	0.040

Besides, a deeper insight into the observed I–V_b characteristics of $MoTe_2/ZrS_2$ and WTe_2/ZrS_2 vdWHs at optimized d has been analyzed in terms of the behavior of the transmission coefficient. The current through the device depends on the transmission amplitude within the bias window of $[\mu_R, \mu_L]$, i.e., $[-V_b/2, V_b/2]$ (see Eqs. (1) and (2)).

The transmission coefficient of $MoTe_2/ZrS_2$ and WTe_2/ZrS_2 are shown in **Figure S5**, the Fermi level is set to be zero and the region of the bias window is $[-V_b/2, V_b/2]$. The $MoTe_2/ZrS_2$ exhibits monotonically increasing I–V_b curves. In this system, the transmission spectra enter more into the bias window with an increase of the applied voltage from 0.5 V to 0.9 V. Consequently, it exhibits a steady rise in current with increasing bias. However, in WTe_2/ZrS_2, as the bias increases from 0.5 V to 0.8 V, the active transmission channels shift away from the most relevant energy region, causing the current decrease from 0.54 μA to 0.34 μA. Upon reaching 0.9 V, a new peak emerges in the transmission spectra, boosting the current increase again.

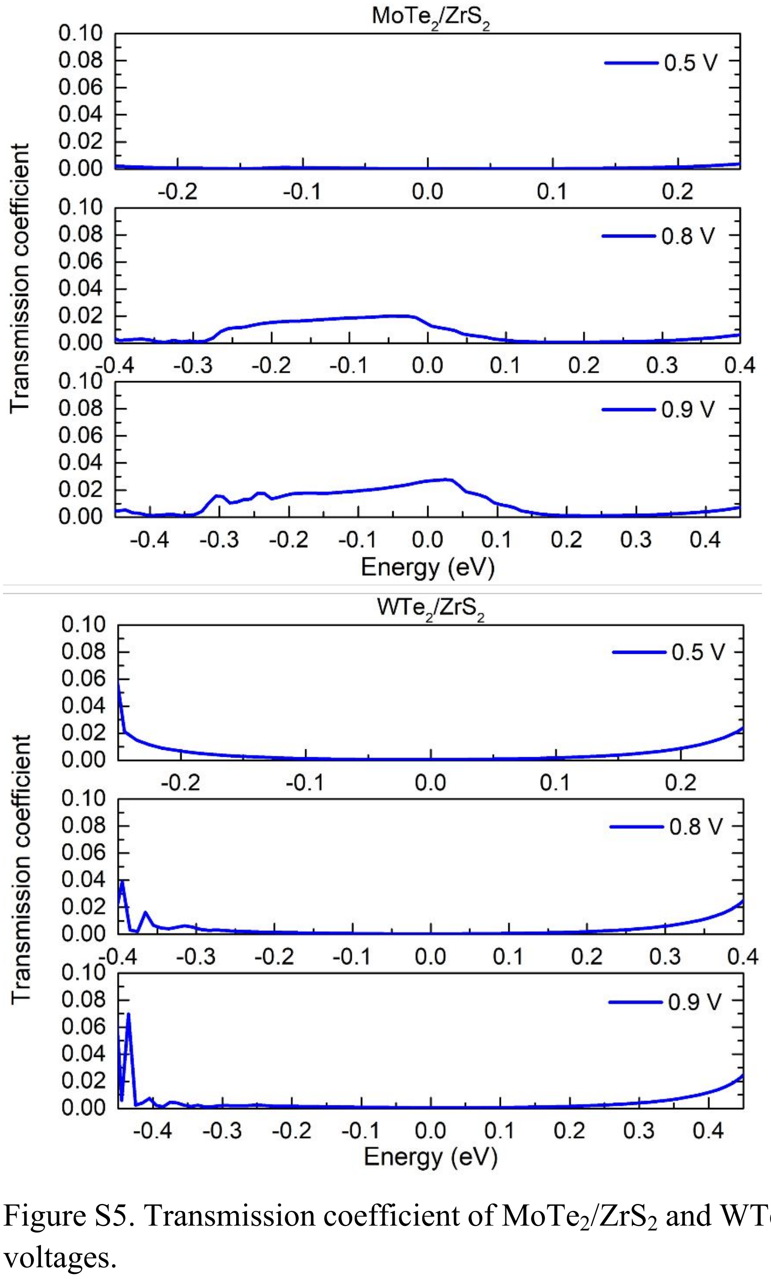

Figure S5. Transmission coefficient of $MoTe_2/ZrS_2$ and WTe_2/ZrS_2 at different applied voltages.

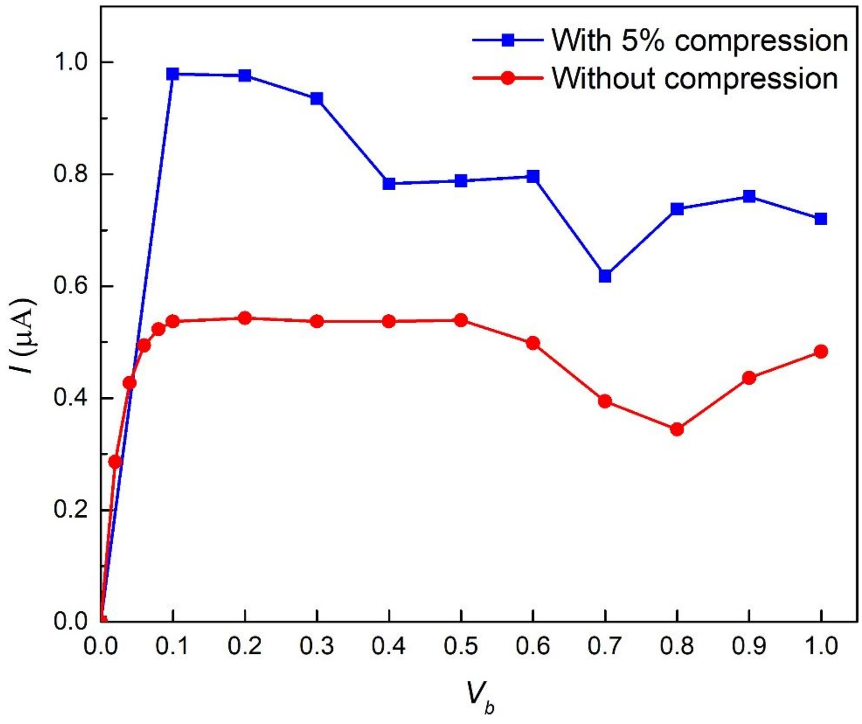

Figure S6. Comparing the effect of lattice mismatch on the I-V_b curve of WTe_2/ZrS_2.

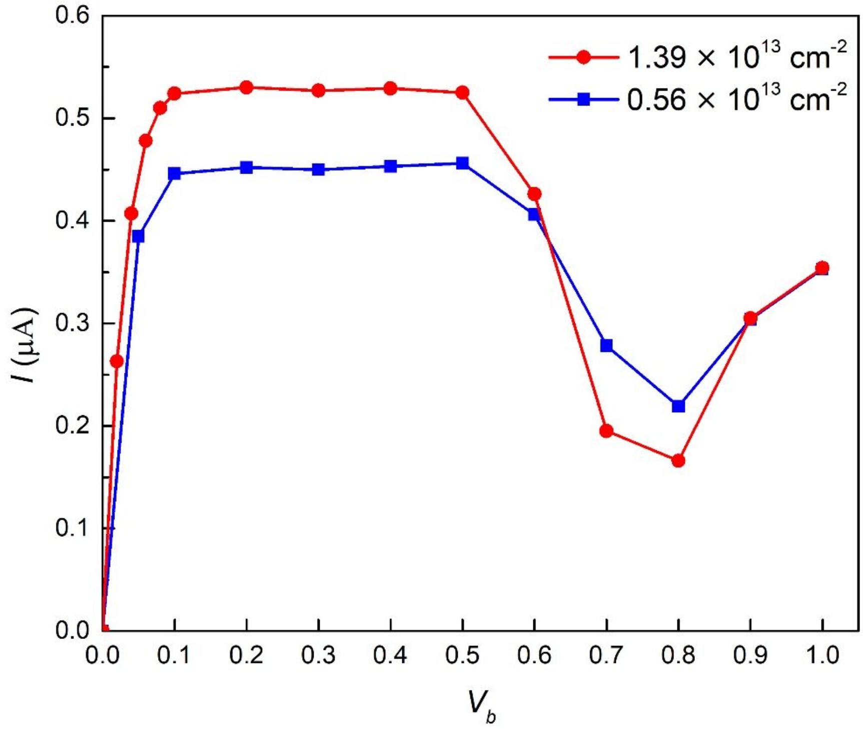

Figure S7. The effect of different p-doped gate effects on the I-V_b curve of WTe_2/ZrS_2.

We note that our calculations do not include the spin-orbit coupling (SOC). This effect will result in the splitting at the valence band maximum (VBM) of TMDs.[16] To see the effect of SOC on the electronic structures of vdWHs, we choose WTe_2/ZrS_2 as a case study. The band structures were calculated using PBE and PBE0, with and without SOC, performed in VASP and displayed in **Figure S8**. When comparing PBE (PBE0) with and without SOC, it is evident that SOC significantly influences the band structure near the K point by splitting the valence band, leading to a slightly more pronounced broken gap. However, while comparing PBE(PBE+SOC) with PBE0(PBE0+SOC), it is found that the band gap is widened with the hybrid functional. Due to error cancellation, PBE and PBE0+SOC exhibit the same extent of broken gap. Considering the accuracy of the band gap and the significantly increased computational demands of using PBE0+SOC, we opted not to include SOC in our primary calculations and used PBE instead. This approach allows us to still conduct a qualitative comparison.

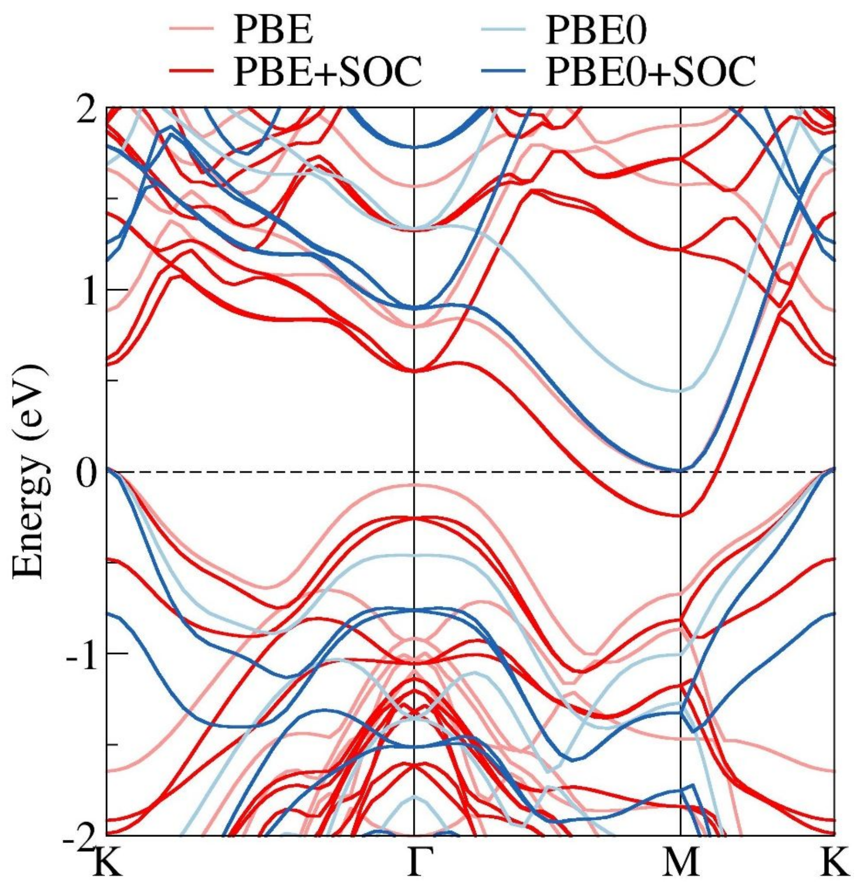

Figure S8. Comparing the effect of soc on the band structure of WTe_2/ZrS_2.

References

(1) Blöchl, P. E., Projector augmented-wave method. *Phys. Rev. B* **1994,** *50* (24), 17953.

(2) Kresse, G.; Joubert, D., From ultrasoft pseudopotentials to the projector augmented-wave method. *Phys. Rev. B* **1999,** *59* (3), 1758.

(3) Kresse, G.; Furthmüller, J., Efficient iterative schemes for ab initio total-energy calculations using a plane-wave basis set. *Phys. Rev. B* **1996,** *54* (16), 11169.

(4) Perdew, J. P.; Burke, K.; Ernzerhof, M., Generalized gradient approximation made simple. *Phys. Rev. Lett.* **1996,** *77* (18), 3865.

(5) Lei, C.; Ma, Y.; Xu, X.; Zhang, T.; Huang, B.; Dai, Y., Broken-gap type-III band alignment in WTe_2/HfS_2 van der Waals heterostructure. *J. Phys. Chem. C* **2019,** *123* (37), 23089-23095.

(6) Guo, Z.; Hu, K.; Su, J.; Chen, J.; Dong, H.; Pan, M.; Nie, Z.; Wu, F., Tunable electronic properties and negative differential resistance effect of the intrinsic type-III ZrS2/WTe2 van der Waals heterostructure. *Appl. Surf. Sci.* **2023,** *611*, 155644.

(7) Soler, J. M.; Artacho, E.; Gale, J. D.; García, A.; Junquera, J.; Ordejón, P.; Sánchez-Portal, D., The SIESTA method for ab initio order-N materials simulation. *J. Condens. Matter Phys.* **2002,** *14* (11), 2745.

(8) Troullier, N.; Martins, J. L., Efficient pseudopotentials for plane-wave calculations. *Phys. Rev. B* **1991,** *43* (3), 1993.

(9) Brandbyge, M.; Mozos, J.-L.; Ordejón, P.; Taylor, J.; Stokbro, K., Density-functional method for nonequilibrium electron transport. *Phys. Rev. B* **2002,** *65* (16), 165401.

(10) Iordanidou, K.; Lara-Avila, S.; Kubatkin, S.; Dash, S. P.; Wiktor, J., Unlocking the Potential of 2D WTe_2/ZrS_2 van der Waals Heterostructures for Tunnel Field-Effect Transistors: Broken-Gap Band Alignment and Electric Field Effects. *Chem. Mater.* **2024,** *36* (22), 11317–11325.

(11) Datta, S., *Electronic transport in mesoscopic systems*. Cambridge university press: Cambridge, England, 1997.

(12) van den Broek, B.; Houssa, M.; Iordanidou, K.; Pourtois, G.; Afanas'ev, V.; Stesmans, A., Functional silicene and stanene nanoribbons compared to graphene: electronic structure and transport. *2D Mater.* **2016,** *3* (1), 015001.

(13) Iordanidou, K.; Wiktor, J., Two-dimensional $MoTe_2/SnSe_2$ van der Waals heterostructures for tunnel-FET applications. *Phys. Rev. Mater.* **2022,** *6* (8), 084001.

(14) Koda, D. S.; Bechstedt, F.; Marques, M.; Teles, L. K., Tuning electronic properties and band alignments of phosphorene combined with $MoSe_2$ and WSe_2. *J. Phys. Chem. C* **2017,** *121* (7), 3862-3869.

(15) Kumar, S.; Schwingenschlogl, U., Thermoelectric response of bulk and monolayer $MoSe_2$ and WSe_2. *Chem. Mater.* **2015,** *27* (4), 1278-1284.

(16) Cheng, Y.; Zhu, Z.; Tahir, M.; Schwingenschlögl, U., Spin-orbit–induced spin splittings in polar transition metal dichalcogenide monolayers. *Europhys. Lett.* **2013,** *102* (5), 57001.
